# Massive Longitudinal Stent Deformation Due to Jailed Atherectomy Wire: When the Problem Is the Solution

**DOI:** 10.1016/j.jaccas.2026.107656

**Published:** 2026-04-10

**Authors:** Amr Mansour Mohamed, Karim Elbasha, Martin Landt, Stephan Fichtlscherer, Holger Nef, Nader Mankerious

**Affiliations:** a Heart Centre, Segeberger Kliniken GmbH, Bad Segeberg, Germany; b Cardiology Department, Faculty of Medicine, Ain Shams University, Cairo, Egypt; c Cardiology Department, Faculty of Medicine, Zagazig University, Zagazig, Egypt

**Keywords:** atherosclerosis, intravascular ultrasound, stents

## Abstract

**Background:**

Longitudinal stent deformation (LSD) is an uncommon complication during percutaneous coronary intervention that has become more frequent with new drug-eluting stents.

**Case Summary:**

We present a case of massive LSD caused by withdrawal of a jailed RotaWire. We could not cross the deformed stent using chronic total occlusion small-balloon and guide-catheter extension support, which left us with a bailout rotational atherectomy through the freshly implanted deformed stent. This resulted in a second complication, namely a burr entrapment.

**Discussion:**

Jailing a RotaWire should always be avoided, and when it inadvertently occurs, wire extraction should be carefully tried after advancing a microcatheter or with predilatation using very small balloons. Once LSD occurs, a stepwise algorithm should be followed to avoid further complication escalation.

**Take-Home Message:**

This case underscores the importance of LSD as a complication during percutaneous coronary intervention and rotational atherectomy; it highlights the possible mechanisms and how to approach it.

## Visual Summary

**Figure:**
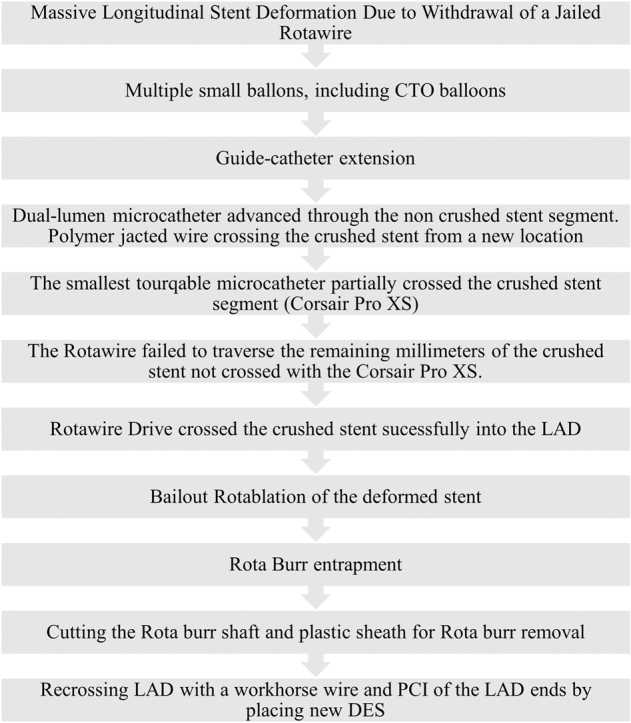

**Visual Summary:**
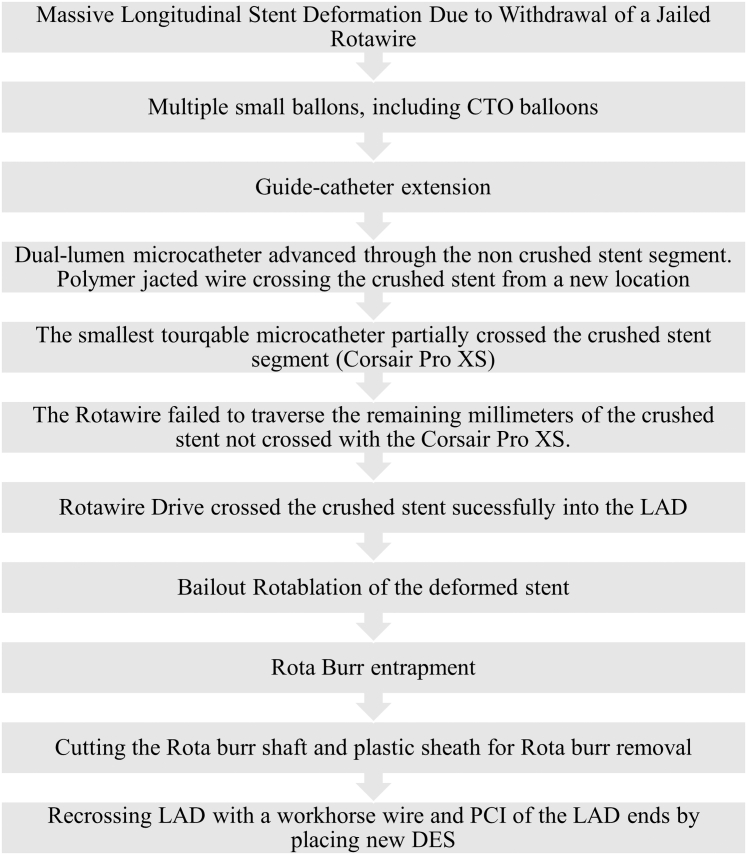
Summary of the Case CTO = chronic total occlusion; DES = drug-eluting stent; LAD = left anterior descending artery; PCI = percutaneous coronary intervention.

New-generation drug-eluting stents (DESs), with thinner struts and fewer connectors, have revolutionized the current era of complex coronary interventions with better flexibility, deliverability, and favorable clinical outcomes; this may be at the expense of longitudinal stent strength.^1^ A small force as low as 0.1 to 1 N can cause longitudinal stent deformation (LSD) in new-generation DESs.^2^^,^^3^ While most recent cases with documented LSD were secondary to mechanical forces caused by inward suction of guiding catheters or guiding catheter extensions during withdrawal of intravascular ultrasound (IVUS) probes, balloons, or other devices,^4^^,^^5^ we describe a case of LSD caused by the withdrawal of a jailed RotaWire (Boston Scientific).

**Take-Home Messages**

• This case highlights the unique features of each wire and catheter used in calcium modification and the risk factors leading to longitudinal stent deformation.
• We should always adopt a stepwise approach for the management of unexpected complications in order to successfully treat them.

## History of Presentation

A 65-year-old man presented for elective percutaneous coronary intervention (PCI) to a mid left anterior descending artery (LAD)–diagonal branch bifurcation lesion (Medina 1.0.1) because of persistent angina pectoris despite optimal medical treatment.

## Intervention

**Figure 1:**
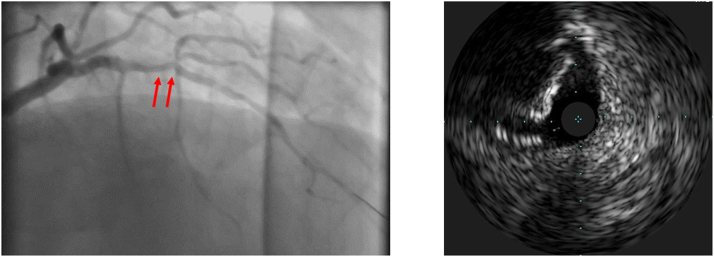
Coronary Angiography and Intravascular Ultrasound (Left) Coronary angiography RAO cranial view shows a significant calcified lesion in the mid-LAD (arrows), and (Right) intravascular ultrasound of the mid-LAD shows a calcified nodule. LAD = left anterior descending artery; RAO = right anterior oblique.

PCI was performed using a right radial approach with a 7-F radial sheath EBU 3.0 guide catheter. IVUS evaluation of the LAD showed a long calcified mid-LAD lesion with a calcified nodule at the origin of the first diagonal branch (Figure 1, Video 1). We opted for upfront elective LAD rotational atherectomy (RA). A RotaWire was exchanged over a Finecross microcatheter (Terumo), followed by 3 runs of RA using a 1.5 burr at 180,000 rpm (Video 2A). A BMW wire was reintroduced, leaving the RotaWire in the LAD as a buddy wire. A second BMW wire was advanced in the diagonal branch. IVUS re-evaluation showed a partially ablated calcified nodule.

**Figure 2:**
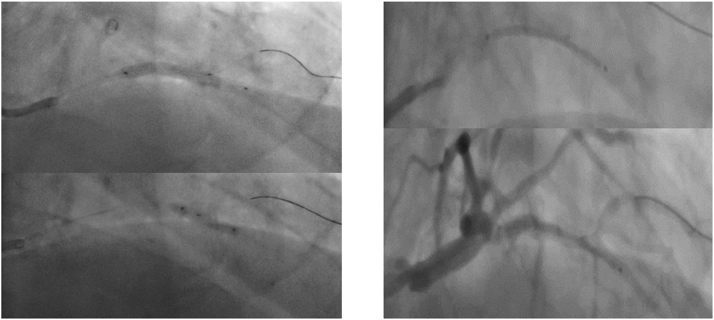
Predilatation and Stent Implantation Angiographic views show lesion preparation and predilatation with (Left) noncompliant balloons and (Right) stent positioning.

**Figure 3:**
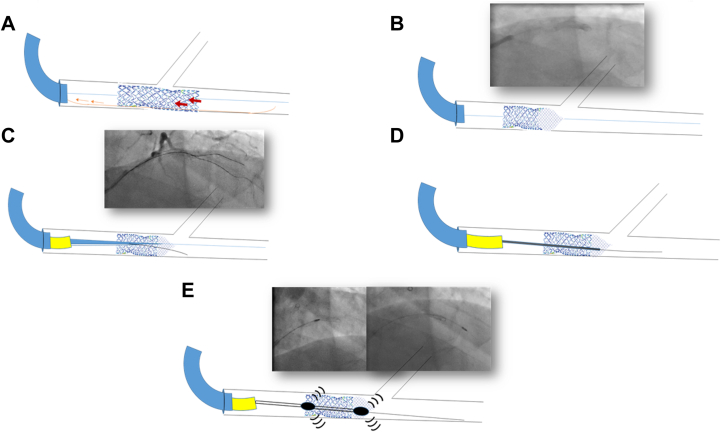
Diagram for Case Management Diagrammatic presentation of the case showing (A) the jailed RotaWire, (B) longitudinal stent deformation and foreshortening, (C) using a dual-lumen microcatheter to pass another wire through another stent strut, (D) partial advancement of a microcatheter through the crushed stent, and (E) rotational atherectomy of the deformed stent.

Sequential predilatation by 2.5- and 3-mm noncompliant balloons was followed by stenting with a 3.5 × 30 mm Onyx TruStar DES (Medtronic) at 12 atm (Figure 2). Unfortunately, we forgot to withdraw the RotaWire before stent deployment, and it was jailed between the stent and the calcified nodule (Figure 3A). Multiple trials were performed to withdraw the RotaWire with application of force and tension while avoiding guiding catheter suction to prevent left main dissection. After successful removal of the RotaWire, a massive LSD with evidence of extreme stent foreshortening was noticed (Figure 3B). This was confirmed by an enhanced stent visualization technique (CLEARstent, Siemens Healthineers), which showed that the distal two-thirds of the stent was deformed into a mesh and metal ball.

At this point, a BMW wire was jailed in the diagonal branch behind the stent struts, and another BMW wire was jailed in the distal LAD through the metal ball. The patient remained hemodynamically stable, without chest pain, and TIMI flow grade III was present in both the LAD and the diagonal branch, allowing sufficient time for further decision-making.

At this time, our complex high-risk indicated-procedure board was convened, and the situation, along with potential solutions, was discussed in the catheterization laboratory.

We tried the following stepwise approach for our current problem:

Our first step was introducing low-profile balloons, including chronic total occlusion (CTO) balloons (1 and 1.2 mm in diameter) through the deformed metal ball over the BMW wire; however, all of them failed to cross the deformed stent.

The second step was increasing our support with a guide catheter extension (Guidezilla, Boston Scientific). However, our smallest available CTO balloon—an 0.85 × 6 mm Sapphire (OrbusNeich Medical)—failed to pass through the stent.

As a third step, we tried to cross the deformed stent from a new location/new strut using a polymer jacketed CTO wire (Fielder XT-A, Asahi Intecc). The wire was advanced through a dual-lumen microcatheter (NHancer, Biotronik), which was introduced over the BMW wire and positioned as distally as possible within the proximal, nondeformed, not well opposed one-third of the stent, to avoid passage of the Fielder wire beneath the proximal stent segment. The Fielder XT-A was manipulated successfully through the side port of the microcatheter into the distal LAD (Figure 3C). We then reattempted to cross the stent with CTO balloons over the Fielder XT-A wire with the additional support from the Guidezilla; however, these attempts were also unsuccessful.

At that point, we were faced with an iatrogenic balloon-uncrossable lesion as a result of a severely crushed stent. We notified our surgical team to be prepared for an urgent bypass operation.

As a last resort, in the fourth step, we attempted to cross the deformed stent as far as possible with the smallest torquable microcatheter, the Corsair Pro XS (Asahi Intecc), which could be partially advanced into the deformed stent segment (Figure 3D). We then exchanged the Fielder XT-A wire with the RotaWire Floppy. However, it failed to cross the remaining few millimeters of the crushed segment. We exchanged this wire with a RotaWire Drive Floppy, as it has better maneuverability, and after multiple trials, it finally succeeded in crossing the deformed stent into the distal LAD.

We removed the diagonal branch wire and performed a bailout RA through the deformed stent using a 1.25-mm burr at 200,000 rpm (Figure 3E). After 2 runs, the burr was able to successfully ablate through the crushed stent (Video 2B). During the third run however, the burr was entrapped in the crushed stent segment.

**Figure 4:**
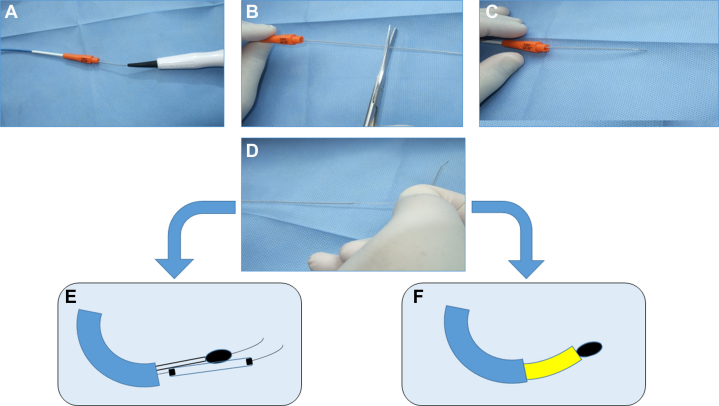
Steps for Management of a Stuck Rota-Burr (A) Separating the burr from the hand, (B) cutting the proximal shaft, (C) removing the plastic sheath, (D) advancing a guide catheter extension, and either (E) removing the stuck burr using a side balloon or (F) deep engaging the guide catheter extension until the burr tip and removing them as a single block

**Figure 5:**
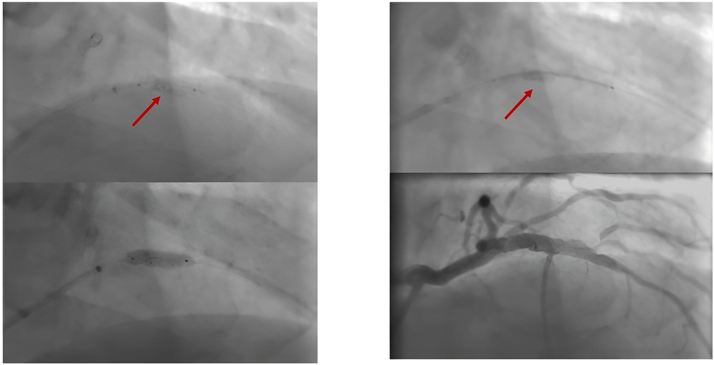
Crushing the Deformed Stent and Implanting a Second Stent Coronary angiogram shows (Left) crushing of the deformed stent using a noncompliant balloon and (Right) stenting through the deformed stent using a second stent.

Several attempts to retrieve the entrapped burr were unsuccessful, and we had to remove it by cutting the drive shaft and removing the plastic sheath, followed by deep advancement of a guide-extension catheter to focus the traction force, allowing in-block extraction of the burr and guidewire as a single unit^6^ (Figure 4). This was followed by introducing a Runthrough wire (Terumo) into the distal LAD and stepwise sequential dilatation of the deformed stent starting with a 1.5 × 6 mm Mini Trek semicompliant balloon (Abbott), followed by upsizing to 2-, 2.5-, and 3-mm noncompliant balloons. Provisional restenting of the LAD was performed with a 3.5 × 30 mm Onyx TruStar DES at 14 atm, followed by proximal optimization technique with a 4 × 12 mm noncompliant balloon, with a good final result (Figure 5).

## Outcome

Follow-up coronary angiography after 3 months showed good results, and the patient was symptom free (Video 3).

## Discussion

New-generation DESs have better profiles and outcomes compared with older generations; however the thinner struts and fewer connectors may result in weaker longitudinal stent strength. Bench studies have described that a minimal force can result in LSD in some new stent platforms.^2^^,^^3^ This force might not be noticed or tacitly felt by operators who do not actively pay attention to the guiding catheter and catheter extensions during advancement and withdrawal of IVUS probes, balloons, and other devices during PCI, leading to sometimes unnoticed LSD. In our case, massive LSD was caused by withdrawal of an inadvertently jailed RotaWire.

Our standard practice is to leave the RotaWire after RA as a buddy wire and withdraw it before stent deployment, as it helps in the delivery of balloons and stents through the heavily calcified and not uncommonly tortuous vessels. The operator, in the present case, forgot to withdraw the RotaWire before stent deployment, resulting in jailing it between the calcium and the stent.

Although jailing percutaneous transluminal coronary angioplasty wire is common during daily PCI practice, especially during complex bifurcation procedures, and is usually inconsequential, this was not the case in our patient.

We think that the extreme LSD in our case was multifactorial, but mainly due to the unique and special design of the RotaWire, which has a shaft thickness of 0.009 inches (0.23 mm) and a tip thickness of 0.014 inches (0.36 mm), unlike other coronary wires, which either have a uniform thickness throughout their length or a tapered distal end. This special design led to a hooking effect of the wire between the distal stent edge and calcified plaque during RotaWire withdrawal and resulted in this massive LSD. Of course, jailing a normal percutaneous transluminal coronary angioplasty wire can also cause LSD.^7^ Other factors that may have contributed to this massive LSD include the presence of a calcified nodule in the mid-LAD lesion, which jailed the RotaWire between the hard calcium and the stent metal. Furthermore, the nonpolymer jacketed coating of the RotaWire might also have been a contributing factor.

**Figure 6:**
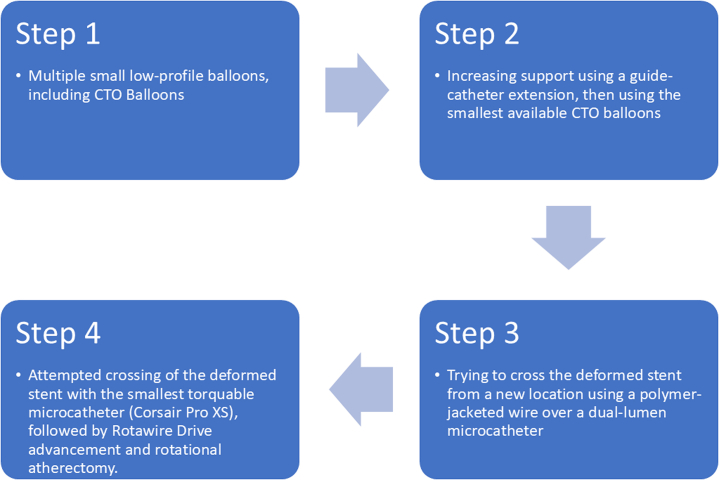
Summary of Our Stepwise Approach to Cross the Deformed Stent CTO = chronic total occlusion.

Similar case reports with LSD caused by a jailed RotaWire were successfully managed by using small balloons directly or with a guide catheter extension and extra support.^4^^,^^5^^,^^7^ These maneuvers and further techniques were not successful in our case, leaving us with one option, which was an off-label RA through a freshly implanted deformed stent (Figure 6).^8^

RA through a freshly implanted stent is not recommended given the higher risk of complications, including slow flow and burr entrapment,^9^ and it should be performed only as an exception in a bailout situation.^6^

## Conclusions

Prevention, early recognition, and proper treatment of LSD during PCI should be performed in a stepwise approach to improve short- and long-term outcomes.

## Funding Support and Author Disclosures

Dr Mankerious has received speaker and consulting honoraria from Teleflex, Biotronik, SIS Medical, and Boston Scientific. All other authors have reported that they have no relationships relevant to the contents of this paper to disclose.

## Appendix

Video 1 Coronary Angiography and IVUS (Left) RAO cranial angiographic view shows a mid-LAD significant calcified lesion, and (Right) IVUS of the mid-LAD shows a calcified nodule.

Video 2 Rotational Atherectomy The video shows lesion preparation using (a) RA and (b) RA of the deformed stent.

Video 3 Control Coronary Angiography Follow-up coronary angiography 3 months later showed stable results.
